# Cost analysis of establishing and operating the first human milk bank at Da Nang Hospital for Women and Children in Vietnam: an activity-based costing ingredients study

**DOI:** 10.1186/s13006-024-00657-6

**Published:** 2024-07-06

**Authors:** Minh V. Hoang, Tuan T. Nguyen, Anh T. Tran, Toan Q. Luu, Mai Q. Vu, Hoang T. Tran, Oanh T. X. Nguyen, Roger Mathisen

**Affiliations:** 1 Hanoi University of Public Health, Hanoi, 11910 Vietnam; 2 Alive & Thrive, Global Nutrition, FHI 360, Hanoi, 11022 Vietnam; 3 Neonatal Unit and Human Milk Bank, Da Nang Hospital for Women and Children, Da Nang, 50506 Vietnam; 4 Department of Pediatrics, School of Medicine and Pharmacy, The University of Da Nang, Da Nang, 50206 Vietnam

**Keywords:** Activity-based costing ingredients (ABC-I), Cost analysis, Donor human milk (DHM), Human milk bank (HMB), Vietnam

## Abstract

**Background:**

Breastfeeding is the biological norm for feeding infants and young children. When mothers’ breastmilk is unavailable, donor human milk (DHM) from a human milk bank (HMB) becomes the next option for small vulnerable newborns. A comprehensive cost analysis is essential for understanding the investments needed to establish, operate, and scale up HMBs. This study aims to estimate and analyze such costs at the first facility established in Vietnam.

**Methods:**

An activity-based costing ingredients (ABC-I) approach was employed, with the cost perspective from service provision agencies (specifically, the project conducted at Da Nang Hospital for Women and Children and Development Partners). Estimated financial costs, based on actual expenditures, were measured in 2023 local currency and then converted to 2023 US dollars (USD). We examined three scenarios: 1) direct start-up costs + indirect start-up costs + implementation costs, 2) direct start-up costs + implementation costs, and 3) capital costs + implementation costs over the 6.5 years of operation.

**Results:**

The total start-up cost was USD 616,263, with total expenditure on direct activities at USD 228,131 and indirect activities at USD 388,132. Investment in equipment accounted for the largest proportion (USD 84,213). The monthly costs of Da Nang HMB were USD 25,217, 14,565, and 9,326, corresponding to scenarios 1, 2, and 3, respectively. Over HMB's 6.5 years of operation, on average, the unit costs were USD 166, USD 96, and USD 62 for DHM received and USD 201, USD 116, and USD 74 for pasteurized DHM meeting specified criteria in the corresponding scenarios. Unit costs were highest in the initial six months, decreased, and reached their lowest levels after a year. Then, the unit costs experienced an increase in late 2020 and early 2021.

**Conclusion:**

Although the unit cost of DHM in Da Nang HMB is comparable to that in certain neighboring countries, intentional measures to reduce disposal rates, improve HMB efficiency, motivate more community-based donors, and establish an HMB service network should be implemented to lower costs.

## Background

Breastfeeding is the biological norm for feeding infants and young children. The World Health Organization (WHO) and UNICEF recommend early initiation of breastfeeding within an hour of birth, exclusive breastfeeding for the first six months of life, and continued breastfeeding with complementary foods for two or more years [1]. Breastmilk is safe and clean and contains antibodies that help protect against common childhood illnesses. Breastfed children perform better on intelligence tests, are less likely to be overweight or obese and are less prone to diabetes later in life. Women who breastfeed also have a reduced risk of breast and ovarian cancers [2].

Suboptimal breastfeeding increases the risk of child mortality, annually resulting in nearly 600,000 deaths among children worldwide [3]. Additionally, it is associated with an annual loss of 21.9 billion liters of breastmilk, equivalent to USD 2.2 trillion, or 10% of the US GDP, 3.7% of APEC’s combined GDP, 12% of the BRICS countries’ combined GDP, 12% of the EU’s combined GDP, and 66% of ASEAN’s combined GDP [4]. In lower- and middle-income countries, the increasing use of complementary feeding for infants aged under 6 months results in emissions of 6.0-7.5 billion kilograms of CO2 equivalent greenhouse gases and 2,562.5 billion liters of water [5].

When mothers’ breastmilk is unavailable, the WHO recommends donor human milk (DHM) from a human milk bank (HMB) as the next option [1, 6]. An HMB is a service that screens, collects, processes, and distributes human breast milk to meet infants' needs for optimal health and development [7]. Donating mothers are recruited, and DHM is collected, stored, processed, and distributed to infants in need [7]. The mission is to supply safe, high-quality DHM to address the needs of infants whose mothers' breastmilk is unavailable. The recipients encompass newborns without access to their mothers' breastmilk, with a particular focus on small vulnerable newborns, including preterm, low birth weight, and sick infants, as well as orphans [8]. The integration of HMBs into health systems is a vital step in ensuring access to safe DHM for infants without access to their mothers’ breastmilk, contributing to a strong start in life [9].

Mothers’ breastmilk and DHM are particularly important for preterm babies because they reduce the incidence of necrotizing enterocolitis (NEC), retinopathy of prematurity, bronchopulmonary dysplasia, and some complications of surgery for congenital heart diseases, brain malformations, thoracic and gastrointestinal abnormalities, or those involving other organs [10, 11]. The use of DHM feeds also helps bridge the gap for newborns temporarily separated from their mothers or when mothers’ breastmilk is not yet available or sufficient [12, 13]. Previous studies have shown that early and exclusive breastfeeding in the first few days of life is critical because it affects exclusive breastfeeding under six months and continued breastfeeding [14, 15].

In Vietnam, before the establishment of the first HMB, the prevalence of low birth weight was 8.2% in 2015, which decreased from 9.2% in 2000. In 2015, although 96.6% of children had ever been breastfed, only 26.5% of babies were breastfed within one hour of birth, and 67.8% of newborns started breastfeeding within one day of birth [16]. The main reason leading to the late initiation of breastfeeding is the insufficient implementation of provider-driven early essential newborn care practices as well as other breastfeeding protection, promotion, and support at health facilities [17–19]. An additional reason is the lack of DHM from an HMB given to newborns when the mother’s breastmilk is unavailable [8, 13], resulting in health facilities stocking and endorsing commercial milk formula and even allowing industry representatives to access the facilities [20]. The establishment of an HMB in Vietnam is critical for providing optimal nutrition for small vulnerable newborns and encouraging the community to view using DHM as the 'new normal' for breastfeeding when mothers’ breastmilk is unavailable.

Additionally, there was a need for the development of national guidelines and the scaling up of the HMB network in Vietnam. A costing study is needed to provide data on the cost of establishing and operating an HMB, as well as the unit cost of providing DHM to eligible children. Furthermore, there is a global scarcity of literature on business cases for investing in HMBs.

To address these needs, this study was conducted to examine the establishment, operation, and unit costs of Da Nang HMB, the first HMB in Vietnam, located at Da Nang Hospital for Women and Children (DNHWC). It considers various scenarios involving financial resources and technical support. The findings of this study have been used to guide the establishment and operation of the Da Nang HMB, as well as four additional HMBs and two HMB satellites, which were primarily established with national technical support.

## Methods

### Study site

Da Nang, a class-1 municipality in Vietnam's central coastal region, spans 1,284.73 square kilometers. It serves as the hub for Central Vietnam's economy, culture, and education, with a population of 1,195,490 and an annual birth rate of 18.68 per thousand. In 2021, Da Nang's infant mortality rate was 8.19 per thousand live births, lower than the national average of 13.65 [21].

DNHWC is a tertiary hospital with 1,200 beds that serves three provinces and has 4.4 million people. The hospital oversees more than 15,000 annual births and specializes in high-risk pregnancies and sick children in the central region. DNHWC is nationally and internationally recognized for its expertise in breastfeeding practices, early essential newborn care, and kangaroo mother care [21].

The Da Nang HMB was established in February 2017. The establishment of this HMB involved various stakeholders, including the Ministry of Health, Da Nang City Department of Health, DNHWC, Alive & Thrive, PATH, and partners and consultants [9]. According to HMB monitoring data, as of July 31, 2023, this HMB had received 11,826 L of donor milk from 586 donors and administered pasteurized donor milk (PDM) to 31,736 newborns over 6.5 years. This HMB served newborns in need in this hospital as well as those in other hospitals in Da Nang city and provided pasteurized DHM to its satellite in Quang Nam province.

### Costing approach

An activity-based costing (ABC) ingredient approach, which combines activity-based costing and ingredient methods, was employed. The ABC method defines the principal activities needed for the program [22, 23]. The ingredient approach involves identifying and costing out all the individual components or ingredients that go into a product or service [24]. We identified the types of inputs, such as building, furniture, equipment, vehicle depreciation, personnel, supplies, water, and electricity costs. We also determined the quantities of the inputs used by the program and the cost per unit of the inputs.

Four main groups of activities related to breastmilk donor involvement, milk handling, processing, milk usage, and general expenses were included in the running phase [9]. More specifically, activities related to breastmilk donor involvement included three processes: screening of donor mothers, selecting donor mothers, and preparing and managing empty containers for donor mothers. The activities related to breastmilk handling and processing included six processes: DHM pasteurization, screening, microbiological testing of pre- and post-pasteurization DHM samples, storage of raw and pasteurized DHM, DHM handling, and transportation. The activities related to DHM usage included two processes: estimating, requesting, and receiving pasteurized DHM and using pasteurized DHM. Finally, activities related to the general operation of the bank included 17 processes: standard of practice (SOP) composition and approval, cleaning and sterilizing instruments, handling HMB instruments, cleaning of functional rooms at the HMB, internal supervision, file storage and writing, detecting and solving incidents, pasteurizer and freezer operation and maintenance, refrigerator maintenance and operation, operation and maintenance of microbiological cultures, operation and maintenance of instrument cleaning, operation and maintenance of a mini autoclave, operation and maintenance of breast pumps, collection and transportation of DHM received from the community, label adherence, and handwashing. In other words, 28 activities were listed for cost calculation in the present study [9].

### Cost perspective

The study's perspective determined which costs were relevant and should be included in the cost analysis, considering who bears the costs [24]. Costs for this first HMB in Vietnam were estimated from the perspective of the service provision agencies, including the HMB project, DNHWC, and other development partners.

### Scope of cost

In this study, we estimated financial costs based on actual expenditures. Expenditure refers to the amount of money spent during a specific period. Costs reflect the resources needed to produce outputs/products during that period [25].

In this study, expenditure and financial costs (accounting costs) were obtained through expenditure reports and all related receipts. Financial costs represent actual expenditures on goods and services purchased. Financial costs were thus described in terms of how much money has been paid for the resources used [24]. This study excluded the opportunity costs incurred by patients and family members.

#### Phase of cost

Both start-up and implementation expenditures/costs were included. Start-up expenditures/costs were resources used for activities implemented between the decision to implement a program and the first time it provided DHM to a small vulnerable newborn [26]. Implementation expenditures/costs were resources used for routine activities.

#### Nature of costs

Both capital and recurrent expenditure/cost items were captured: Capital expenditures were one-time expenses to acquire assets, which were normally expensive (e.g., building, furniture, equipment, vehicles). Recurrent costs, including personnel, supplies, and operational expenses such as water and electricity, were incurred within a single financial or accounting year.

#### Function of costs

**Fig. 1:**
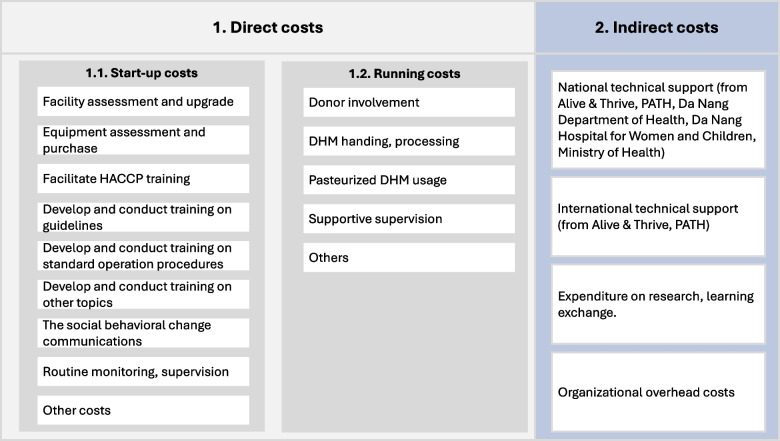
Costs included in this study

Both direct and indirect costs were measured. Direct costs were resources used for implementing the activities directly linked to the process of service delivery (donor involvement; DHM collection, handling, processing, and usage; and general operational expenses); indirect costs were resources used for activities that could not be attributed to a particular program, such as overhead and information, education, and communication activities. The costs included in this study are presented in Fig. 1.

### Data collection

The data were collected in 2016 and 2017 by a research team from the Center of Population Health Science, Hanoi University of Public Health. The team reviewed activity and expenditure reports to extract data on start-up activities and expenditures. For each activity, we identified costs by location and financial ingredient (e.g., staff cost, materials, supplies, transportation, building, equipment).

Field visits to DNHWC were conducted twice to collect data on implementation activities. In-depth interviews with HMB staff and direct observation of HMB procedures were performed during these field visits to calculate implementation costs.

### Data management and analysis

The data were entered into a computer using Excel spreadsheets. Costs were presented in Vietnamese dong (VND) for expenses in Vietnam or in US dollars (USD) for expenses overseas. All costs were measured on June 15, 2023, in local currency units and converted to 2023 US dollars at an exchange rate of 1 USD = 23,881 VND, as announced by the State Bank of Vietnam [27].

The total monthly cost was estimated in two steps: Step 1 involved allocating expenditure from the start-up phase to the monthly start-up cost using useful years and the inflation rate, and step 2 involved adding the monthly implementation cost to the monthly start-up cost.

The unit cost is calculated as 1) cost per liter of pasteurized DHM that is suitable for use (i.e., meeting standards such as pre- and post-pasteurization tests, physiological appearance, and within expiration dates) and 2) cost per liter of DHM expressed by mothers (raw breastmilk) for international comparison purposes.

Three cost scenarios were examined (Table 1):

- Scenario 1: Total costs (direct start-up costs + indirect start-up costs + implementation costs).
- Scenario 2: Total direct costs (direct start-up costs + implementation costs).
- Scenario 3: Total processing cost (capital costs + implementation costs).

**Table 1:** Three cost scenarios

**Cost items**	**Scenario 1**	**Scenario 2**	**Scenario 3**
**Direct costs**			
**Start-up phase**			
Facility assessment and upgrade	√	√	√
Equipment assessment and purchase	√	√	√
Facilitate HACCP training; develop guidelines; develop and conduct training on SOPs; training on HMB procedure; social & behavior change communication; routine monitoring (development of forms and electronic system); others (general expenses)	√	√	
**Running phase**			
Donor involvement; DHM handling, processing; DHM usage; others (general expenses)	√	√	√
**Indirect costs (start-up phase only)**			
National technical support (from A&T, PATH, DOH, DNHWC, and MOH)	√		
International technical support (from A&T, PATH), research, and learning exchange	√		

*A&T* Alive & Thrive, *DOH* Da Nang Department of Health, *MOH* Ministry of Health, *DHWCH* Da Nang Hospital for Women and Children, *HACCP* hazard analysis and critical control points, *SOPs* standards of practice

## Results

### Startup costs

The start-up phase occurred from January 2015 to the end of January 2017. The total start-up cost of the HMB project was USD 616,263 (Table 2). The total expenditure on direct activities was USD 228,131, and that on indirect activities was USD 388,132. Investment in equipment accounted for the largest proportion (USD 85,775).

**Table 2:** Total start-up expenditure of the Da Nang HMB by activity group

**Activity group**	**Value** (2023 USD)	**%**
**Direct activities group**	**228,131**	**37.0**
Facility assessment and upgrade	24,270	3.9
Equipment assessment and purchase	85,775	13.9
Facilitate HACCP training with the HMB team	3,467	0.56
Guidelines development, advocacy, and approval	14,070	2.3
Develop and train on SOPs	603	0.10
Training on breastfeeding promotion, donor recruitment, and education; monitoring and reporting	50,199	8.1
SBCC activities^a^	27,336	4.4
Routine monitoring (including development of forms and electronic system), supervision, meetings	8,140	1.3
Other: Dissemination meetings/events, advocating for HMB, obtaining approval from DOH and MOH	14,271	2.3
**Indirect activities group**	**388,132**	**63.0**
Research (e.g., formative research, costing study, and feeding study)	5,176	0.8
Learning exchange	58,189	9.4
National technical support	174,519	28.3
International technical support	150,249	24.4
**Total**	**616,263**	**100.0**

*HACCP* hazard analysis and critical control points, *SOPs* standards of practice, *SBCC* social and behavioral change communication

^a^SBCC activities include 1) development, pretesting, and finalization; development, pretesting, and finalization of posters, video clips, leaflets, donor ID cards, and mascot; printing/reprinting materials; 2) branding; and 3) opening ceremonies

### Implementation costs

#### Total monthly cost of the Da Nang HMB

As shown in Table 3, the monthly cost of Da Nang HMB based on the first scenario (direct start-up costs + indirect start-up costs + implementation costs) was USD 25,217. The monthly cost based on the second scenario (direct start-up costs + implementation costs) was USD 14,565, and the monthly cost based on the third scenario (capital costs + implementation costs) was USD 9,326.

**Table 3:** Monthly costs of Da Nang HMB by activity group

Activity group	Scenario 1		Scenario 2		Scenario 3	
	Value	%	Value	%	Value	%
*Unit: 2023 US dollars*						
**Direct activities group at the start-up phase**	**6,469**	**25.7**	**6,469**	**44.4**	**1,230**	**13.2**
Facility assessment and upgrade	174	0.7	174	1.2	174	1.9
Equipment assessment and purchase	1,056	4.2	1,056	7.3	1,056	11.3
Facilitate HACCP training	627	2.5	627	4.3		
Guidelines	440	1.7	440	3.0		
Training on SOPs	253	1.0	253	1.7		
Training on breastfeeding promotion, donor recruitment & education, and monitoring & reporting	1,675	6.6	1,675	11.5		
SBCC	498	2.0	498	3.4		
Routine monitoring	437	1.7	437	3.0		
Other: Administrative, meeting, advocacy, approval	1,310	5.2	1,310	9.0		
**Indirect activities group at the start-up phase**	**10,651**	**42.2**				
Research	1,144	4.5				
Learning exchange	644	2.6				
National technical support	4,847	19.2				
International technical support	4,016	15.9				
**Implementation costs**	**8,096**	**32.1**	**8,096**	**55.6**	**8,096**	**86.8**
Donor involvement	1,368	5.4	1,368	9.4	1,368	14.7
DHM handling, processing	3,456	13.7	3,456	23.7	3,456	37.1
DHM usage	2,037	8.1	2,037	14.0	2,037	21.8
Operations and maintenance of equipment; management & supervision	1,235	4.9	1,235	8.5	1,235	13.2
**Total**	**25,217**	**100.0**	**14,565**	**100.0**	**9,326**	**100.0**

*HACCP* hazard analysis and critical control points, *SOPs* standards of practice, *SBCC* social and behavioral change communication; routine monitoring: development of forms and electronic systems

Scenario 1: direct start-up costs + indirect start-up costs + implementation costs; scenario 2: direct start-up costs + implementation costs; scenario 3: capital costs + implementation costs

#### The breakdown of the monthly cost

**Fig. 2:**
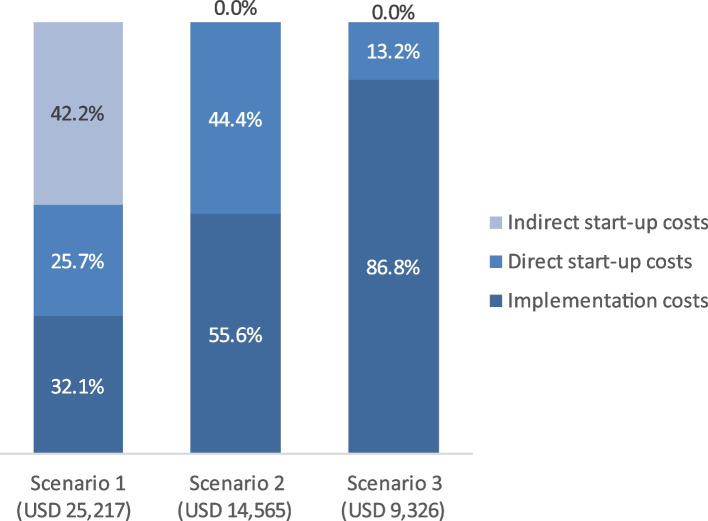
Composition of the monthly cost of HMB based on different scenarios. Scenario 1: direct start-up costs + indirect start-up costs + implementation costs; scenario 2: direct start-up costs + implementation costs; scenario 3: capital costs + implementation costs

Figure 2 presents the composition of the monthly cost of HMB based on the three scenarios. For scenario 1 (including all costs), start-up costs accounted for the largest share (67.9%), of which 25.7% were for direct activities and 42.2% were for indirect activities. Implementation costs made up 32.1% of the monthly cost of HMB. With scenario 2 (excluding indirect costs from scenario 1), direct start-up costs accounted for 44.4%. Related activities in the implementation phase made up 55.6% of the total HMB cost per month. Finally, scenario 3 (excluding training, guideline development, routine monitoring, and the SBCC) reveals that direct start-up costs, presented as capital costs only, accounted for 13.2% of the total cost per month. Related activities in the implementation phase made up 86.8% of the total HMB cost per month.

**Fig. 3:**
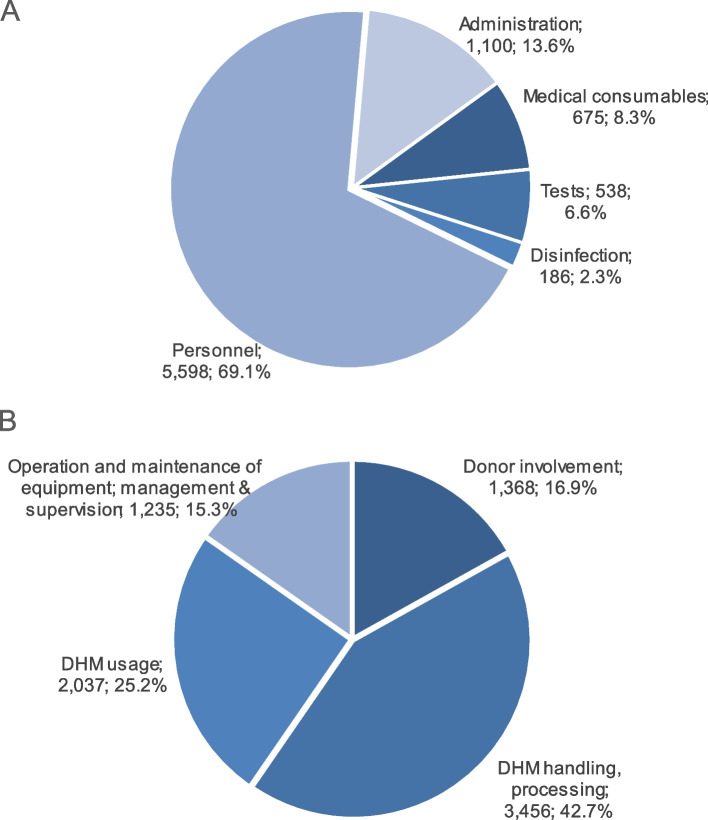
Breakdowns of the monthly implementation expenditure by ingredients (**A**) and activity group (**B**) out of a total of USD 8,096. The data are presented as USD, %

Figure 3 presents the breakdowns of the monthly implementation expenditures by ingredient and activity group. The expenditures on personnel and administration work made up considerable shares of the total monthly implementation expenditure (69.1% and 13.6%, respectively). The expenditures on medical consumables, tests, and disinfection accounted for 17.2% of the total expenditure.

DHM handling and processing accounted for the largest share (42.7%), followed by DHM usage (25.2%) of the total implementation expenditure (Fig. 3). Expenditures on activities related to donor involvement and to donor involvement, equipment operation and maintenance, management, and supervision accounted for 16.9% and 15.3%, respectively, of the total implementation expenditures (Fig. 3).

#### Unit cost

**Fig. 4:**
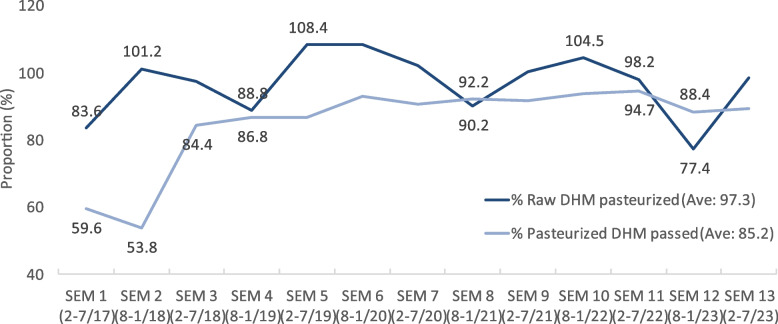
The percentage of DHM pasteurized and pasteurized DHM passed pre- and post-screening tests. SEM: semester, every six months

In the initial 12 months of operation, the proportion of pasteurized DHM that passed both pre- and post-pasteurization tests was low in the first (59.6%) and second (53.8%) six months (denoted as semesters) (Fig. 4). Subsequently, it increased significantly to a range of 84.4% to 94.7% (Fig. 4). The percentage of DHM used for pasteurization started at 83.6% in the first semester, increased to 101.2% in semester 2 and then fluctuated between the lowest value of 77.4% in semester 12 and highest values of 108.4% in semester 5 (Fig. 4).

**Fig. 5:**
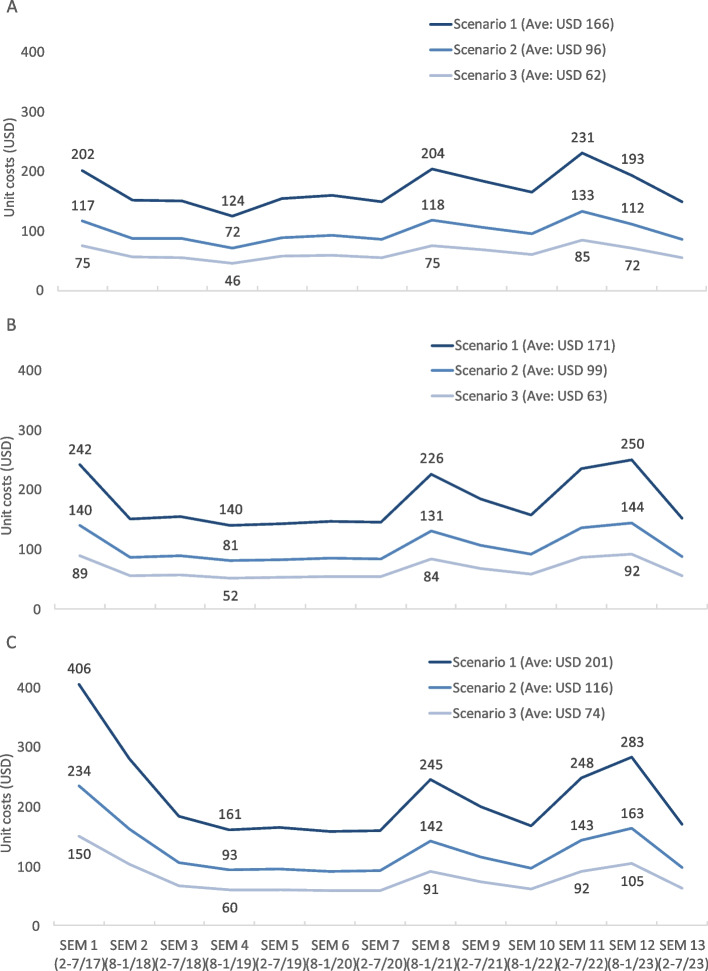
The cost (USD) of each liter of raw breastmilk (**A**), breastmilk pasteurized (**B**), and pasteurized DHM met the pre- and posttest criteria (**C**). SEM: semester, every six months. Scenario 1: direct start-up costs + indirect start-up costs + implementation costs; scenario 2: direct start-up costs + implementation costs; scenario 3: capital costs + implementation costs

Over the 6.5 years of operation, the average costs for DHM received in its raw form were USD 166, USD 96, and USD 62 for scenarios 1, 2, and 3, respectively (Fig. 5). The corresponding costs for pasteurized DHM meeting the specified criteria were USD 201, USD 116, and USD 74. However, these values exhibited variation over time. Costs were highest in the initial six months, decreased, and reached their lowest levels after one year. Subsequently, values experienced an increase in late 2020 and early 2021 (Fig. 5).

## Discussion

### The cost of establishing Da Nang HMB is high

This was the first costing study of an HMB in Vietnam, which was the first facility established in the country. We found that the total expenditure on the Da Nang HMB in 2023, at USD 616,263 (under scenario 1, with all costs included), is higher than the USD 225,000 reported in a study conducted in Australia in 2012 [28]. Because this was the first HMB, there was limited national experience. The involvement of international experts, overseas learning exchanges, and attending conferences to build networks added costs. Furthermore, conducting formative research, feeding studies, and designing a monitoring system are expensive and require external experts. In addition, the Da Nang HMB applied high standards and was even more conservative than European HMBs [9]. For example, after formative research identified DHM safety as the greatest concern, Da Nang HMB decided to pool DHM from a single donor, which made it easier to track any problems. This requirement necessitated at least three large freezers, which increased costs. Additionally, applying 'operation room' standards to design the storage and processing room for DHM further increased costs. The Da Nang HMB also purchased a laminar flow hood to provide a sterile working area for milk processing before pasteurization and for splitting milk from containers into smaller portions to distribute to recipients. For the first HMB, additional expenses were needed to advocate for and inform communities about the HMB [15]. If indirect activities were not included (i.e., scenario 3), the total expenditure of the Da Nang HMB (USD 228,131) would be similar to the figure reported by this Australian study of USD 200,000–250,000 [28].

### Including various scenarios would help to identify financial needs in various settings

Scenario 1 entails the costs of the first HMB in settings with limited national experience and heavy dependence on external expertise. Additionally, research is needed to direct the operation of HMB or generate knowledge. Scenario 2 would apply to settings with strong national expertise (e.g., from the second HMB in Vietnam), even though national guidelines may not be available. Scenario 3 can be used for the scaling-up period of HMB networks under the guidance of experienced national experts and national guidelines. In the remainder of this article, we discussed scenarios 2 and 3 because they are more appropriate for the routine establishment of an HMB.

### The average cost per liter of pasteurized donor milk at Da Nang HMB tends to be lower than that in other countries

The cost per liter of pasteurized DHM ready to be used (i.e., passed both pre- and post-pasteurization tests) over 6.5 years in our study was USD 116 (scenario 2) and USD 74 (scenario 3), corresponding to an overall milk disposal rate of approximately 15%. The cost per liter of DHM varies across countries and HMB [29–37], from the lowest of 41.4 USD in an HMB in China [37] to 370 USD in an HMB in the UK [34]. In the UK, the estimated cost of 1 L of DHM was reported to be approximately GBP 150–290 or USD 191–370 in a position paper that did not specify the sources of information [34]. The unit cost in our study was higher than that reported in a study in China, which was just USD 41 per liter [37]. Several possible reasons may account for this difference, including 1) our HMB has a longer period of operation (eight years), 2) most donors are not required to repeat the needed tests when donating within six months of their serological test, and 3) raw DHM from all mothers on the same day is brought to pasteurization [37]. In contrast, Da Nang HMB pools raw DHM from each donor, thus needing to store raw milk until the volume reaches approximately eight liters or more. This practice requires more funds to buy freezers, allocate space for them, and allocate resources for running and operating [9].

However, due to potential variations in operating procedures and costing methodologies, the findings might not be readily comparable. HMBs in Norway have a cost of USD 100 per liter; however, they use raw milk and primarily serve newborns at the same hospital, which can reduce the cost of establishing and operating an HMB [33]. In Spain, a study compared high-temperature short-time (HTST) pasteurization (72-75 °C, 15 s) and traditional Holder Pasteurization (HoP) (62 °C, 30 min) for donor milk. Despite the high initial equipment investment required for HTST pasteurization, this process leads to a substantial long-term reduction in production costs [31].

### Staffing remains the main cost

In this study, we found that staffing remains the main monthly cost: almost 70% of implementation costs and 60% when start-up costs are included. The cost was higher than that reported in other studies applying similar methodologies: 33% in China, 40% in Italy, and 51% in Germany [30, 32, 36]. The study collected information about task distribution in the first seven months of HMB, when operations and staff performance might not be the most efficient. In addition, the issues relating to the high disposal rate of milk would surely require additional efforts for investigation, discussion, and solution development. Moreover, the staff of Da Nang HMB play various roles, including clinical roles, while non-HMB staff clinicians and nurses also provide support to HMB (e.g., donor recruitment, education, and feeding of newborns). It is possible that our study misclassified the costs related to personnel incorrectly. Nonetheless, we believe that the personnel cost could be lower if it is measured later in the implementation stage.

### Unit costs vary with time

In this study, we found that the cost was greater in the initial period. The disposal rate was high (up to almost 50%), leading to a high volume of DHM that could not be used, which increased the unit cost. Additionally, we observed that pasteurization of only 84% of the raw DHM occurred within the first six months. Da Nang HMB might need to wait until the volume reaches at least 8 L before pasteurization, delaying the process and resulting in a lower volume of pasteurized DHM and thus a higher unit cost. In subsequent years, the cost decreased due to the lower disposal rate (less than 15%). Starting in the second year, the Da Nang HMB also began recruiting more donors from the community who contributed larger volumes of breastmilk for an extended duration [12], resulting in a lower disposal rate [38] and helping to reduce costs.

However, we identified two periods when unit costs increased, regardless of the disposal rate. The first peak occurred between August 2020 and January 2021 and was linked to the COVID-19 pandemic and included travel restrictions, fear of transmission, hesitancy to contact others, and limitations on hospital visits unless necessary. At times, DNHWC was quarantined due to suspected COVID-19, leading to a decreased supply of DHM and reduced use of pasteurized DHM. The second peak in unit cost occurred from February 2022 to January 2023, corresponding to a period in which the percentage of raw DHM pasteurized dropped due to the high volume of pasteurized DHM at the HMB in August 2021-July 2022 (approximately 300-350 L) compared to approximately 200 L or less after July 2022. Additionally, there was a decrease in the volume of raw DHM. Due to challenges in procurement, the hospital could not perform all four screening tests for donors (HIV, HBV, HCV, and syphilis) simultaneously, resulting in the inability to recruit certain potential donors.

The change in unit cost over time suggests that new HMBs might need 6-12 months to optimize their operation. Our findings also propose that a high volume of raw DHM received, a low disposal rate, and a high usage of pasteurized donor milk would lower the unit cost. Therefore, instead of having multiple HMBs, having HMB satellites would be a more cost-effective investment. The satellites collect raw DHM and send it to the HMB for processing, receiving pasteurized DHM for their newborns in need. This helps reduce the cost of establishing an HMB.

### Scaling up of the HMB model in Vietnam

For the effective replication of this model to serve multiple regions in Vietnam (i.e., reducing the cost of DHM), four additional HMBs and two HMB satellites have been established based on national expertise. Core staff from the established HMBs support facility assessments and capacity building for newer HMBs. Using the Resource Toolkit for Establishing and Integrating Human Milk Bank Programs [7] and drawing on 100 years of international learning in processing and providing DHM through HMBs [8], Vietnam has developed its own HMB guidelines [39] for future scaling up of HMB networks. Furthermore, an HMB network in the East Asia Pacific has been established to facilitate knowledge sharing among countries in the region. An HMB standard for the East Asia Pacific was issued with the contributions of all member states [40]. National HMB experts from the network also participate in the development of global guidance on HMB.

This study has several limitations. This approach utilizes the establishment cost of the first HMB, whose components might be essential for other HMBs (e.g., scenario 1). As mentioned earlier, the inclusion of three scenarios addresses various needs of HMB establishment. Additionally, costs will vary depending on operational procedures, which might differ across HMBs. Implementation costs are based on the first seven months of operation, which might differ from subsequent phases that could be more efficient (e.g., requiring less effort from staff and having better operational procedures).

## Conclusion

This study offers a comprehensive estimation of financing for the first HMB in Vietnam. While the cost of DHM in this study is comparable to that of certain neighboring countries, it is considered high in comparison to that of other HMBs with a long duration of operation since its establishment. Measures aimed at achieving better quality control, minimizing the loss of DHM volume, and motivating more community-based donors to contribute breastmilk to the HMB, as opposed to relying solely on hospital donors, should be intentionally implemented. These efforts are crucial for reducing costs and ensuring that access to this service is both available and sufficient for eligible small vulnerable infants whose mothers’ breastmilk is unavailable.

## Data Availability

Not applicable. An Excel file for the costing estimation will be available from the corresponding author upon request.

## Abbreviations

ABC-I: Activity-based costing ingredients
DHM: Donor human milk
DNHWC: Da Nang Hospital for Women and Children
HMB: Human milk bank
NEC: Necrotizing enterocolitis
