# Correction: Cost analysis of establishing and operating the first human milk bank at Da Nang Hospital for women and children in Vietnam: an activity-based costing ingredients study

**DOI:** 10.1186/s13006-024-00658-5

**Published:** 2024-07-29

**Authors:** Minh V. Hoang, Tuan T. Nguyen, Anh T. Tran, Toan Q. Luu, Mai Q. Vu, Hoang T. Tran, Oanh T. X. Nguyen, Roger Mathisen

**Affiliations:** 1 Hanoi University of Public Health, Hanoi, 11910 Vietnam; 2 Alive & Thrive, Global Nutrition, FHI 360, Hanoi, 11022 Vietnam; 3 Neonatal Unit and Human Milk Bank, Da Nang Hospital for Women and Children, Da Nang, 50506 Vietnam; 4 Department of Pediatrics, School of Medicine and Pharmacy, The University of Da Nang, Da Nang, 50206 Vietnam

**International Breastfeeding Journal (2024) 19:47**

10.1186/s13006-024-00657-6

Following publication of the article, it came to the authors’ attention that the last sentence of the Conclusion section was incorrect. Namely, it read as follows: “These efforts are crucial for reducing costs and ensuring that access to this service is not available or sufficient for eligible small vulnerable infants whose mothers’ breastmilk is not available.” Whereas it should be as follows: “These efforts are crucial for reducing costs and ensuring that access to this service is both available and sufficient for eligible small vulnerable infants whose mothers’ breastmilk is unavailable.”

The published article has been updated to fix this error. The authors thank you for reading this erratum and apologize for any inconvenience caused.

